# The carbohydrate-active enzymes database (CAZy) in 2013

**DOI:** 10.1093/nar/gkt1178

**Published:** 2013-11-21

**Authors:** Vincent Lombard, Hemalatha Golaconda Ramulu, Elodie Drula, Pedro M. Coutinho, Bernard Henrissat

**Affiliations:** ^1^Centre National de la Recherche Scientifique, CNRS UMR 7257, 13288 Marseille, France and ^2^Aix-Marseille Université, AFMB, 163 Avenue de Luminy, 13288 Marseille, France

## Abstract

The Carbohydrate-Active Enzymes database (CAZy; http://www.cazy.org) provides online and continuously updated access to a sequence-based family classification linking the sequence to the specificity and 3D structure of the enzymes that assemble, modify and breakdown oligo- and polysaccharides. Functional and 3D structural information is added and curated on a regular basis based on the available literature. In addition to the use of the database by enzymologists seeking curated information on CAZymes, the dissemination of a stable nomenclature for these enzymes is probably a major contribution of CAZy. The past few years have seen the expansion of the CAZy classification scheme to new families, the development of subfamilies in several families and the power of CAZy for the analysis of genomes and metagenomes. This article outlines the changes that have occurred in CAZy during the past 5 years and presents our novel effort to display the resolution and the carbohydrate ligands in crystallographic complexes of CAZymes.

## INTRODUCTION

Despite their similar chemical composition, carbohydrates can form an enormous number of combinations through the stereochemical variety of the hydroxyl groups that they carry, through the many possibilities to assemble monosaccharides one to another, and through the wealth of noncarbohydrate substituents that can decorate the resulting oligo- and polysaccharides. Complex carbohydrates are widely distributed in nature, where they mediate a multitude of biological functions, from carbon reserve, to structural molecules, or as the mediators of intra- and intercellular recognition within one organism or between organisms. The diversity of complex carbohydrates is controlled by a panel of enzymes involved in their assembly (glycosyltransferases) and their breakdown (glycoside hydrolases, polysaccharide lyases, carbohydrate esterases), collectively designated as Carbohydrate-Active enZymes (CAZymes). CAZymes have been classified in sequence-based families for >22 years (1–6) and this classification has become the standard of the field (7).

The first defining feature of CAZyme classification is that the families are defined based on significant amino acid sequence similarity with at least one biochemically characterized founding member (1). The consequence is that sequences that display too little similarity to ensure a significant alignment are not included, nor used to form putative families, as distant relatives of CAZymes may have other functions. Borderline cases are stored in the nonclassified section of each CAZyme category, awaiting biochemical characterization. A second defining feature is that our classification is made module by module. CAZymes are frequently modular proteins with a catalytic module harbouring a variable number of other discrete modules, which can be either catalytic or not. Thus a modular CAZyme can be assigned to several families if its constitutive modules belong to separate families. The third important feature is that we only analyse systematically protein sequences released in the daily releases of GenBank (ftp://ftp.ncbi.nih.gov/genbank/daily-nc), to avoid analysing unfinished sequences that may change accession number.

As early as 1991, it was noted that the sequence-based families of glycoside hydrolases grouped together enzymes of different substrate specificities (i.e. enzymes with ‘different’ EC numbers) (1) demonstrating that the acquisition of novel specificity has been commonplace during evolution. This feature was subsequently noted for the other classes of CAZymes (4,6). The processes by which a novel substrate specificity was acquired from a common ancestor leave detectable traces in the sequence of contemporary proteins. Thus, unexpectedly, the usual drawback of carbohydrates (their chemical resemblance) is at the origin of their success in the postgenomic era: CAZymes need to be specific to perform their biological functions. While the precise specificity of DNAses, RNAses, proteases and esterases is difficult or impossible to derive from their sequence alone, the CAZyme classification system allows in some cases the prediction of the broad category of carbohydrate substrate, based on the assignment to a family (8). This carries the potential to infer the glycobiological profile of an organism (or a community thereof) based on DNA sequence. However, the occurrence of enzymes that act on different substrates in the same family is a significant problem for the automated functional annotation of CAZyme-related genes. This can sometimes be overcome by the definition of subfamilies within families (9,10) (see below), but our current knowledge of the sequence-to-specificity relationships in CAZymes families is still largely insufficient and unevenly distributed for many families to allow unsupervised automated substrate prediction.

The Carbohydrate-Active Enzymes database (CAZy; http://www.cazy.org) was launched in 1999 to provide online and constantly updated access to the family classification of CAZymes. Coupled to the CAZypedia encyclopaedic resource (http://www.cazypedia.org), CAZy is the only comprehensive resource that correlates the sequence, structure and molecular mechanism of CAZymes. CAZy was presented in this journal in 2009 (11) and the present article outlines the changes that have been implemented in CAZy during the past 5 years.

## WEBSITE DESIGN

**Figure 1.**
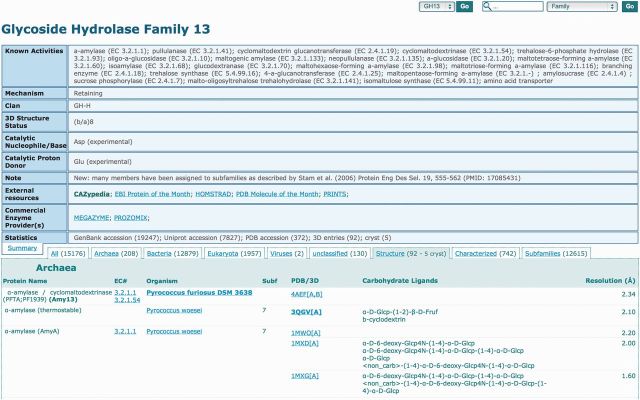
A view of the GH13 page showing the newly available 3D structural information (carbohydrate ligands and resolution) in the Structure tab.

In March 2011, the website interface was deeply redesigned both in appearance (new layout, new colours and new logo) and in content. Thus new sections and new links have been added to commercial providers that list their products following the CAZy nomenclature. Other additions cover scientific meetings relevant to CAZymes, positions available and a ‘what’s new’ section that provides news on changes in the CAZy database. More interactivity in the display of information associated with each family was introduced (Figure 1). In particular, each family has now a specific tab, which lists those individual CAZymes that we believe have been experimentally characterized. Because the number of entries in several families had become impractical, the display was modified to just show the header for each family along with a series of tabs for access to subsets (All, Archea, Bacteria, Eukaryota, unclassified, Structure, Characterized). Each tab displays 1000 entries per page, except for the tab listing the characterized enzymes, where only 100 entries are shown per page. The search tool was also revisited and one can now search the entire site or specific fields such as CAZy family, taxonomic identifier, organism name, protein name, accessions in different databases (GenBank, Uniprot and Protein Data Bank (PDB)), known activities, EC number, mechanisms or clan.

## NOVEL ENZYME CLASS

Because lignin is invariably found together with polysaccharides in the plant cell wall and because lignin fragments are likely to act in concert with polysaccharide lytic mono-oxygenases (LPMO), families of lignin degradation enzymes and of LPMOs have been used to define a new CAZy class that we have named ‘Auxiliary Activities’ to accommodate a broad range of enzyme mechanisms and substrates related to lignocellulose conversion (12).

## DATABASE GROWTH

At the date of submission of this article, CAZy reports sequence information on almost 340 000 CAZymes, a staggering 225% increase compared with 5 years ago (Table 1). During the same period, the number of biochemically characterized CAZymes has grown by only 30% to 12 700 and the number of CAZymes with 3D structures has grown by ∼78% (Table 1). Despite this growth, only ∼1400 (0.4%) of the 340 000 CAZymes have a 3D structure solved to date. The past 5 years have seen the number of families covered by CAZy grow slowly to >330 at present. Five years ago the number of genome sequences analysed in CAZy was 750 (11). This number is now greater than 2800 (see below), representing a 3.8-fold increase. The continuously growing gap between the number of sequences and the number of biochemically or structurally characterized CAZymes is a direct consequence of the avalanche of genome sequences resulting from modern sequencing technologies combined with the much lower pace of experimental characterization of gene products. This gap would even be more considerable if one was to search and list CAZymes in nonfinished genomes.

**Table 1.** Growth of the CAZy database during the past 5 years

Protein class	Sequences Sept-2013	Dec-2008	Characterized Sept-2013	Dec-2008	With structure Sept-2013	Dec-2008
GH	159 274	46 654	9221	6805	817	475
GT	119 910	40 863	1936	1846	139	83
PL	4043	1301	336	262	51	34
CE	15 856	5083	275	212	74	43
CBM	32 259	9210	663	570	280	166
AA	5801	464^a^	299	71^a^	58	3^a^
Total	337 143	103 111	12 730	9695	1419	801

^a^Numbers estimated from the literature: the AA category did not exist in December 2008.

## DATABASE CONTENT: SUBFAMILIES

The occurrence of enzymes that act on different substrates in the same family prevents the straightforward functional annotation of CAZyme-related genes. The division of CAZyme families into subfamilies based on phylogenetic analysis has been explored as a possible approach to improve the relationship between sequence and specificity. Subfamily classification of GH5, GH13, GH30 and all of the PL families has shown that the majority of the defined subfamilies are monospecific, thus indicating that the correlation of substrate specificity with sequences is significantly better at the subfamily level than the family level (9,10,13). An additional benefit of the division into subfamilies is the identification of currently uncharacterized subfamilies that can subsequently be analysed experimentally to unveil potential new activities. Subfamilies are currently displayed for families GH5, GH13, GH30, AA1–AA5 and for all PL families. Many more families are currently evaluated for subfamily definitions. Care is taken that the subfamilies are defined in a robust manner to avoid confusion that would arise from constant redefinitions and resulting different naming conventions. We prefer to let the subfamilies ‘mature’ until we feel that the subfamily quality and stability is sufficient for public release.

## DATABASE CONTENT: GENOMES

The collection of carbohydrate-active enzymes encoded by the genome of an organism (**‘**CAZome**’**) provides an insight into the nature and extent of the metabolism of complex carbohydrates of the species. The CAZomes of free-living organisms typically correspond to 1–5% of the predicted coding sequences. Extremely reduced CAZomes are characteristic of species with a strict intracellular parasitic lifestyle. Because of the massive chemical, structural and functional variability of carbohydrates, CAZome comparisons can highlight the adaptation of the CAZymes repertoire of species to their environment (14,15).

Since 2011, in addition to giving the family distribution, the new CAZy website displays the complete list of putative CAZymes (with accession numbers) of each genome that was analysed. At present, CAZy covers >2800 genomes in the following kingdoms: Bacteria (2351), Archea (158), Eukaryota (73), Viruses (240). The CAZomes listed in the CAZy website correspond to protein models of finished genomes, i.e. with proteins released in the daily releases of GenBank (ftp://ftp.ncbi.nih.gov/genbank/daily-nc). In a few cases, genomes with protein models not released as finished entries in GenBank but publically available, have been analysed and are presented in CAZy. However, for these few cases, the display only shows the number of proteins in each family, but does not feature the actual list of proteins.

Genomes are analysed using the CAZy pipeline, which combines Blast and HMM tools to compare protein models, respectively, with sequence and profile libraries created from the sequences of the catalytic and noncatalytic modules of the CAZy database. This is followed by a manual inspection by expert curators to resolve borderline cases (11). Our methodology provides coherent, expert and comparable sets of annotations. In this respect, one should note that the correspondence between CAZy families and those in PFAM (16)/INTERPRO (17) or DBCAN (18) is far from perfect. This is due to a variety of reasons that include different strategies, different thresholds, different goals, different methods, different training sets and different degrees of expert curation. An unfortunate consequence is that the CAZyme analysis of a genome performed with one method usually cannot be compared with that done with another.

There are two ways to get a genome analysed by CAZy: if the genome and encoded proteins are deposited as finished entries in GenBank (or EMBL or DDBJ) they will be analysed by our daily routines. Alternatively, if one wishes to perform a CAZy analysis before deposition to GenBank (or EMBL or DDBJ), one should approach us for collaboration. Metagenomic data are analysed exclusively in collaboration due to their usual large size.

## DISPLAY OF STRUCTURAL INFORMATION

The CAZy database is not only used by those who wish to analyse genomes, but also by structural biologists who study the molecular details of substrate recognition by CAZymes. Until September 2013, the only information available in the structure pages of CAZy was the accession and macromolecule chain name(s) in the PDB (http://www.rcsb.org) (19). We have made a series of developments to provide additional information relevant to the 3D structure of CAZymes such as the resolution (for crystal structures) and a description of the carbohydrate ligands found in the CAZyme binding sites.

The resolution information is straightforward to generate, as it is present in the PDB files of structures solved by x-ray crystallography. When the resolution information is unavailable in the PDB file, the type of experimental method by which the structure was solved is given instead (powder diffraction or nuclear magnetic resonance).

On the other hand, the PDB does not provide any option to perform a comprehensive search for carbohydrate structures found in CAZyme binding sites and, unlike proteins or nucleic acids, the nomenclature for carbohydrate residues within PDB files is not standardized (20). In addition, the information on how the isolated carbohydrate residues are linked to each other is not described in PDB files. We thus extract the carbohydrate ligand information from PDB files using PDB-care (http://www.glycosciences.de/tools/pdb-care/) (21,22). The carbohydrate molecules covalently linked to an Asn or a Ser/Thr residue were discarded to eliminate N- and O-glycans to identify the carbohydrate ligands bound to CAZyme active sites. The latter are shown in the structure pages of CAZy following their IUPAC nomenclature.

Not all carbohydrate structures are susceptible to automated description by PDB-care. In a number of cases, we have manually curated and provided IUPAC descriptions for structures that are unsuitable to PDB-care: (i) nonreducing glycans (cyclodextrins, sucrose and sucrose derivatives, trehalose, kestose, raffinose, nystose, etc.), (ii) ligands that contain both carbohydrate and noncarbohydrate moieties such as acarbose and acarbose derivatives, (iii) sulfur-containing oligosaccharides, (iv) fluorine-containing carbohydrates and (v) oligosaccharides containing 3,6-anhydro bridges. Table 2 displays examples of the manually handled cases. In addition, automated scripts have been devised to handle ∼180 carbohydrate analogues that we denote <carb_like_ligandref> where ligandref corresponds to the three-letter ligand name given by the PDB. For instance, the carbohydrate-like inhibitor 1-deoxynojirimycin appears as <carb_like_NOJ>. The structural biology community is invited to contact us to report the possible errors that might have slipped through our curation process.

**Table 2.** Examples of carbohydrate ligands treated manually

Category	Common name	Display in CAZy structure pages	Example of PDB file
Nonreducing oligosaccharides	α-cyclodextrin	α-cyclodextrin	3EDF
	β-cyclodextrin	β-cyclodextrin	3CGT
	Sucrose	α-D-Glcp-(1-2)-β-D-Fruf	4FFH
	Raffinose	α-D-Galp-(1-6)-α-D-Glcp-(1-2)-β-D-Fruf	1W2T
	Kestose	α-D-Glcp-(1-2)-β-D-Fruf-(1-2)-β-D-Fruf	3LDR
	Nystose	α-D-Glcp-(1-2)-β-D-Fruf-(1-2)-β-D-Fruf-(1-2)-β-D-Fruf	3LEM
Thio-oligosaccharides	Thio-cellobiose	β-D-Glcp-(1-4)-β-D-Glcp4S	4IPM
	Thio-laminaribiose	β-D-Glcp-(1-3)-β-D-Glcp3S	1J8V
	Thio-xylopentaose	β-D-Xylp-(1-4)-β-D-Xylp4S-(1-4)-β-D-Xylp4S-(1-4)-β-D-Xylp4S-(1-4)- β-D-Xylp4S	3CUJ
	α-methyl-thio-cellopentaoside	β-D-Glcp-(1-4)-β-D-Glcp4S-(1-4)-β-D-Glcp4S-(1-4)-β-D-Glcp4S-(1-4)- α-D-Glcp4S-(1-1)-methyl	1H5V
Fluoro-oligosaccharides	5-fluoro-β-D-glucose	β-D-Glcp5F	4AMX
	2-deoxy-2-fluoro-α-D-glucose	α-D-Glcp2F	1UYQ
	5-fluoro-β-D-xylose	β-D-Xylp5F	2XVK
3,6-anhydro oligosaccharides	Neoagarohexaose	α-L-3,6-anhydro-Galp-(1-3)-β-D-Galp-(1-4)-α-L-3,6-anhydro-Galp-(1-3)- β-D-Galp-(1-4)-α-L-3,6-anhydro-Galp-(1-3)-β-D-Galp	2CDO
	Porphyran/agarose hexasaccharide	α-L-Galp6SO3-(1-3)-α-D-Galp-(1-4)-α-L-3,6-anhydro-Galp-(1-3)- β-D-Galp-(1-4)-α-L-Galp6SO3-(1-3)-α-D-Galp	4AW7
	Agarooctaose	α-L-3,6-anhydro-Galp-(1-3)-β-D-Galp-(1-4)-α-L-3,6-anhydro-Galp-(1-3)- β-D-Galp-(1-4)-α-L-3,6-anhydro-Galp-(1-3)-β-D-Galp-(1-4)- α-L-3,6-anhydro-Galp-(1-3)-β-D-Galp	4ATF
Acarbose and its derivatives	Acarbose	<non_carb>-(1-4)-α-D-6-deoxy-Glcp4N-(1-4)-α-D-Glcp-(1-4)-β-D-Glcp	3ZOA
	Acarbose-derived trisaccharide	<non_carb>-(1-4)-α-D-6-deoxy-Glcp4N-(1-4)-α-D-Glcp	1XCW
	Acarbose-derived pentasaccharide	α-D-6-deoxy-Glcp4N-(1-4)-α-D-Glcp-(1-4)-<non_carb>-(0-4)- α-D-6-deoxy-Glcp4N-(1-4)-α-D-Glcp-(1-4)-β-D-Glcp	1PIG

As of September 2013, >1400 CAZymes and modules thereof have a known 3D structure, corresponding to almost 6000 PDB entries out of which ∼1500 carbohydrate (or carbohydrate analogue) ligands are now identified and presented in the structure tab of each CAZy family.

## FUTURE DIRECTIONS

CAZy is a knowledge-based resource that aims to link the sequence, the specificity and the 3D structural features of CAZymes. How these enzymes achieve selective recognition of target substrates that display only subtle stereochemical differences is key to prediction of substrate specificity. While this is already achievable for a few subfamilies, we are still a long way from a reliable automated substrate (and/or product) prediction for all CAZymes encoded by a genome. We believe that subfamily-based target selection for experimental investigation of CAZymes will progressively fill the knowledge gap that will allow reliability in future functional predictions.

## FUNDING

Agence Nationale de la Recherche, grant BIP:BIP [ANR-10-BINF-03-04]. Funding for open access charge: Waived by Oxford University Press.

*Conflict of interest statement*. None declared.
